# Iron influence on dissolved color in lakes of the Upper Great Lakes States

**DOI:** 10.1371/journal.pone.0211979

**Published:** 2019-02-13

**Authors:** Patrick L. Brezonik, Jacques C. Finlay, Claire G. Griffin, William A. Arnold, Evelyn H. Boardman, Noah Germolus, Raymond M. Hozalski, Leif G. Olmanson

**Affiliations:** 1 Department of Civil, Environmental, and Geo- Engineering, University of Minnesota, Minneapolis, MN, United States of America; 2 Department of Ecology, Evolution, and Behavior, University of Minnesota, St. Paul, MN, United States of America; 3 Remote Sensing Laboratory, Department of Forest Resources, University of Minnesota, St. Paul, MN, United States of America; University of Maryland Center for Environmental Science, UNITED STATES

## Abstract

Colored dissolved organic matter (CDOM), a major component of the dissolved organic carbon (DOC) pool in many lakes, is an important controlling factor in lake ecosystem functioning. Absorption coefficients at 440 nm (*a*_440_, m^-1^), a common measure of CDOM, exhibited strong associations with dissolved iron (Fe_diss_) and DOC in 280 lakes of the Upper Great Lakes States (UGLS: Minnesota, Wisconsin, and Michigan), as has been found in Scandinavia and elsewhere. Linear regressions between the three variables on UGLS lake data typically yielded R^2^ values of 0.6–0.9, suggesting that some underlying common processes influence organic matter and Fe_diss_. Statistical and experimental evidence, however, supports only a minor role for iron contributions to *a*_440_ in UGLS lakes. Although both DOC and Fe_diss_ were significant variables in linear and log-log regressions on *a*_440_, DOC was the stronger predictor; adding Fe_diss_ to the linear *a*_440_-DOC model improved the R^2^ only from 0.90 to 0.93. Furthermore, experimental additions of Fe^III^ to colored lake waters had only small effects on *a*_440_ (average increase of 0.242 m^-1^ per 100 μg/L of added Fe^III^). For 136 visibly stained waters (with *a*_440_ > 3.0 m^-1^), where allochthonous DOM predominates, DOM accounted for 92.3 ± 5.0% of the measured *a*_440_ values, and Fe_diss_ accounted for the remainder. In 75% of the lakes, Fe_diss_ accounted for < 10% of *a*_440_, but contributions of 15–30% were observed for 7 river-influenced lakes. Contributions of Fe_diss_ in UGLS lakes to specific UV absorbance at 254 nm (SUVA_254_) generally were also low. Although Fe_diss_ accounted for 5–10% of measured SUVA_254_ in a few samples, on average, 98.1% of the SUVA_254_ signal was attributable to DOM and only 1.9% to Fe_diss_. DOC predictions from measured *a*_440_ were nearly identical to those from *a*_440_ corrected to remove Fe_diss_ contributions. Overall, variations in Fe_diss_ in most UGLS lakes have very small effects on CDOM optical properties, such as *a*_440_ and SUVA_254_, and negligible effects on the accuracy of DOC estimated from *a*_440_, data for which can be obtained at broad regional scales by remote sensing methods.

## Introduction

Research associating iron (Fe) concentrations and organic color (now called colored dissolved organic matter, or CDOM) in surface waters extends back to studies in Finland [1] and Sweden [2], but its nature and significance were poorly understood for many decades. CDOM plays a major role in the ecological functioning of lakes by affecting light penetration, temperature structure, metal bioavailability, and photochemical processes. Several recent studies, e.g., [3,4], have implicated Fe as a factor in the long-term increases observed in CDOM across Scandinavia [5, 6] and some other temperate regions–the so-called “browning” phenomenon [7]. Increasing total Fe (Fe_T_: dissolved plus particulate Fe) in 27 of 30 Swedish rivers was estimated to account for an average of 25% of the variations in CDOM and up to 74% in northern Sweden [2]. Ekström et al. [8] proposed that long-term CDOM trends in Swedish rivers could be related to increasing Fe mobilization driven by increasing temperature and river discharge that increase the probability of anoxic conditions conducive to Fe solubilization.

Whether the Fe-CDOM relationship is actually causative or merely correlative may affect the use of CDOM, which can be retrieved on regional scales from satellite imagery, e.g., [9,10], to estimate concentrations of DOC, a major component in the aquatic carbon cycle. If Fe affects absorption coefficients (*a*_λ_) at the wavelength (λ) used to quantify CDOM, variations in dissolved Fe or in the fraction of *a*_λ_ caused by Fe could affect the accuracy of DOC estimated from *a*_λ_. Here we address this issue for lakes in the U.S. Upper Great Lakes States (UGLS).

Most recent studies on the influence of Fe on CDOM have focused on Swedish lakes. Based on observations from multi-basin Lake Mälaren, Köhler et al. [11] found decreasing dissolved Fe (Fe_diss_) as water flowed through the basins, with concurrent declines in CDOM and a shift from colored allochthonous material to less colored autochthonous DOM. Weyhenmeyer et al. [4] found a linear relationship between dissolved organic carbon (DOC) and CDOM (measured as absorption coefficients at 420 nm, *a*_420_) in a large dataset from Sweden and Canada, but the carbon-specific *a*_420_ (*a*_420_/DOC) increased nonlinearly, approaching an asymptotic value, with increasing Fe_T_, which the authors considered to be all Fe_diss_. Based on these findings, the authors inferred that Fe_diss_ affected apparent CDOM levels (i.e., absorption coefficients at 420 nm, *a*_420_) and concluded that Fe_diss_, pH, water residence time, and colored DOC all may be important factors for regional changes in lake browning. Alternative explanations for the browning phenomenon, including climate change [12] and recovery from acidification by atmospheric acid deposition, e.g., [5,13], are not necessarily inconsistent with a role for Fe.

Effects of Fe on UV absorbance are well studied, but effects in the visible range are less well known. Weishaar et al. [14] found that absorbance at 254 nm (A_254_) increased with Fe^III^ at the same rate in solutions with or without DOM. Poulin et al. [15] found A_254_ increased linearly with Fe^III^ in DOM-containing solutions but found no effect for added Fe^II^. They concluded that Fe^III^ should be accounted for in measurements of specific UV absorbance at 254 nm (SUVA_254_; i.e., A_254_ normalized by DOC) and provided an equation to make such corrections. Maloney et al. [16] found a nonlinear increase in carbon-specific absorptivity, *a*_320_/DOC in the Fe_diss_ range of 1–4 mg/L in a humic-rich lake and reported that the spectral slope in the range 280–400 nm decreased as Fe_diss_ increased from 0.0 to 0.5 mg/L. They hypothesized that Fe_diss_ likely would affect light conditions in the visible range but made no measurements in this region. Kritzberg and Ekström [3] and Xiao et al. [17]) reported that adding Fe^III^ to CDOM-containing waters linearly increased absorptivity at 410–420 nm. Adding Fe^III^ (1600–3600 μg/L) to humic and fulvic acid reference materials also decreased spectral slopes in the UV range [17].

We have been studying characteristics of CDOM in UGLS lakes and mapping its distribution by field studies and satellite imagery [10,18,19]. The occurrence of major iron ore deposits in Minnesota led us to question whether Fe contributes to observed CDOM levels (measured as *a*_440_) and/or affects its other optical properties, and whether that could affect DOC values inferred from *a*_440_. This study had three primary objectives: (1) quantify the association between Fe and *a*_440_ (our measure of CDOM) in surface waters of three UGLS ecoregions; (2) experimentally determine whether the association is causal, and if so evaluate the extent of Fe contributions to *a*_440_ and other optical properties that characterize CDOM; and (3) quantify the influence of Fe variability on DOC estimated from *a*_440_ in natural waters.

## Methods

### Sampling sites

**Fig 1:**
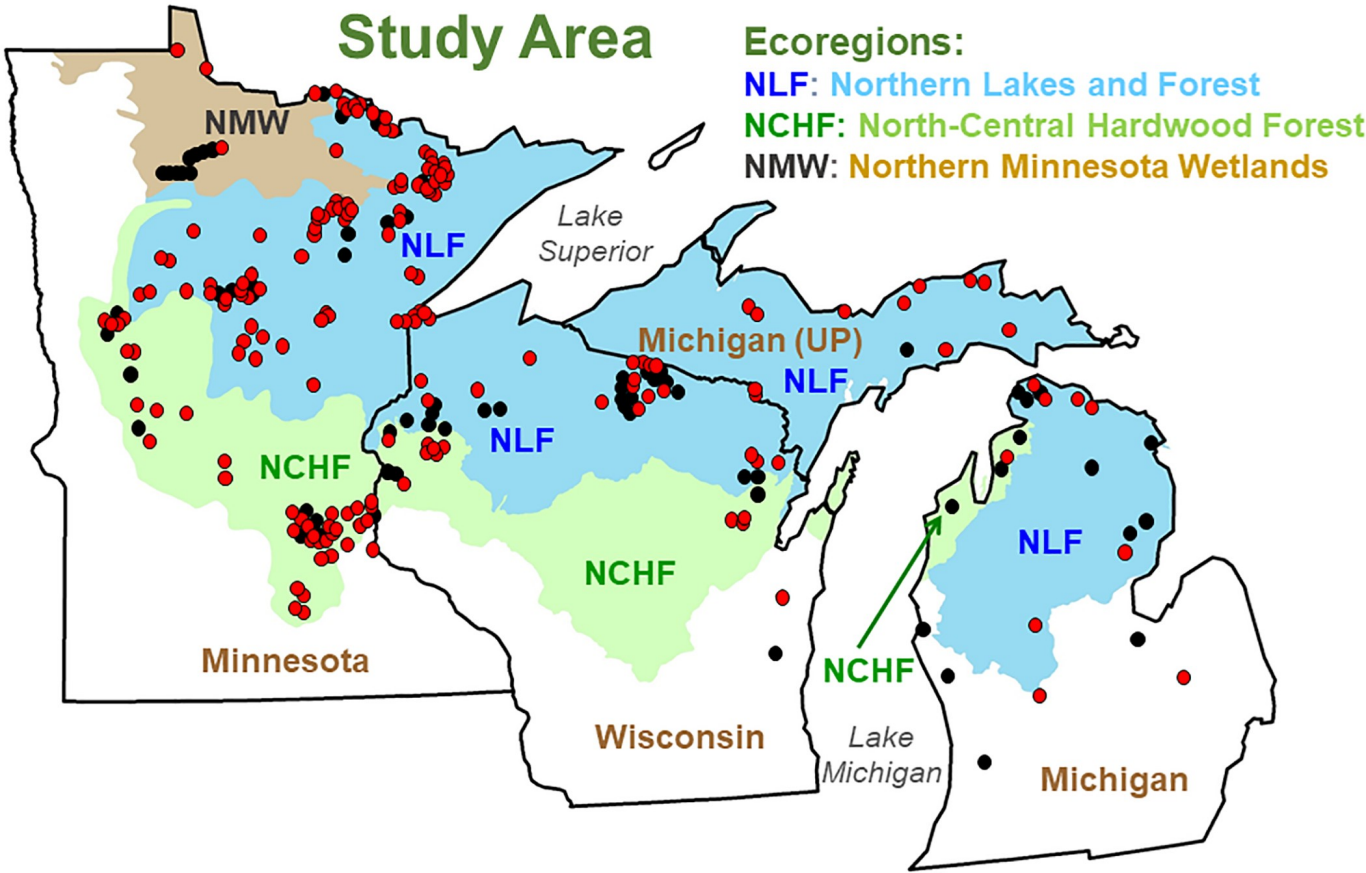
Map of study area showing ecoregions and sampling sites. Red circles: sites with Fe_diss_, *a*_440_ and DOC data; black circles: additional sites with only *a*_440_ and DOC data.

We collected 450 samples from 280 water bodies (mostly lakes) in northern and central Minnesota, Wisconsin, and Michigan over the period 2014–2018 (Fig 1). Sampling occurred during summer (June-September). Nearly all 2014 and 2015 samples were from two lake-rich ecoregions of northeastern and east-central Minnesota: Northern Lakes and Forest (NLF) and North-Central Hardwood Forest (NCHF), which together contain ~ 9800 of the state’s ~12,000 lakes. The NLF is ~ 50% forested, and nearly a third of its area is wetlands or lakes. Agriculture and urban land cover constitute only small portions of this ecoregion (7 and 4%, respectively). In contrast, the NCHF is ~ 48% agricultural land and 9% urban. Forest cover constitutes 25% and wetlands ~ 10% of the NCHF. In 2016, sampling was extended to NLF and NCHF areas in Wisconsin and Michigan and the Northern Minnesota Wetlands (NMW) ecoregion, which has < 200 lakes, only a few of which are road-accessible.

Water usually was collected by small boat or kayak in the open water area, but collections were made from the ends of docks on small lakes where boat access was not feasible. Sites were selected to include a diversity of lake types, CDOM levels, and catchment land cover. The vast majority of sites were lakes, but six large rivers and six impoundments on large rivers were included. NLF lakes were in forested catchments (mixed conifers and hardwoods with substantial wetlands) with little to no human development; supplemental sampling in 2018 focused on the NLF ecoregion, where the vast majority of CDOM-rich lakes occur in the study area. Some NCHF lakes were in minimally developed catchments, but most were in urban to exurban areas and a few had catchments with row-crop agriculture.

### Sampling/Field procedures

Water samples were collected from ~ 0.25 m depth using acid-washed, triple-rinsed polycarbonate or high-density polyethylene bottles and stored on ice until processed, usually the same evening. Secchi depth (SD) was measured by standard limnological procedures. Samples were collected at various depths on a few NLF lakes in 2018 to determine effects of stratification on CDOM and Fe_diss_. Raw water was filtered through 0.45 μm Geotech trace-metal-certified capsule filters or pre-combusted (4 h at 450 ^o^C) 0.7 μm Whatman glass fiber filters. Filtered water for DOC and Fe_diss_ analyses was acidified using 0.1 mL of 2 M HCl per 50 mL of sample and refrigerated (DOC) or frozen (Fe_diss_) in pre-cleaned glass or plastic bottles, respectively. Unfiltered water for Fe_T_ analyses was acidified with 1 mL of concentrated HNO_3_ per 50 mL sample and stored in the same manner as the Fe_diss_ samples. Un-acidified filtered water for CDOM analysis was refrigerated in 40 mL glass vials with no headspace. Filter blanks (DI water) showed no measurable DOC or CDOM. Chlorophyll-*a* (chl-*a*) was collected by vacuum filtration of water samples onto 0.22 μm cellulose nitrate filters that were then stored frozen until analysis.

### Analytical methods

Absorbance was measured within a month of sample collection by scanning from 250 to 700 nm using a Shimadzu 1601UV-PC dual beam spectrophotometer with 1 cm or 5 cm quartz, depending on CDOM levels, and nanopure water in the reference cell. We tested whether length of storage affected *a*_440_ measurements on filtered, refrigerated samples from three colored lakes with *a*_440_ values of 5–30 m^-1^ and found no detectable decreases in *a*_440_ after one month of storage, which agrees with other studies [20]. Samples were allowed to warm to room temperature on the benchtop prior to measurements. Absorbance was converted to Napierian absorption coefficients using:

$$a_{\lambda }=2.303A_{\lambda }/l$$ (1)

where: *a*_λ_ is the absorption coefficient (m^-1^) and A_λ_ is absorbance, both at wavelength λ, and *ℓ* is cell path length (m). Absorbance was blank-corrected before conversion. CDOM is reported as absorption coefficient (m^-1^) at 440 nm, *a*_440_. SUVA_254_ (L mg^-1^ m^-1^) was calculated by dividing absorbance at 254 nm by DOC concentration (mg/L), after correcting for cell path length. Contributions of Fe_diss_ to SUVA_254_ were calculated using the equation of Poulin et al. [15]; subtraction of the Fe_diss_ contribution yielded DOM-based values, SUVA_254,DOM_. Spectral slopes (*S*_λ2-λ1_) were calculated from absorbance data for three wavelength regions (275–295, 350–400, and 400–460 nm) by taking the natural logarithm (ln) of A and computing slopes in Excel or by nonlinear fit of absorptivity data to Eq (2):

$$a_{\lambda }=a_{\lambda ,ref}exp\{-S(\lambda -\lambda_{ref})\}$$ (2)

where λ_*ref*_ is a reference wavelength and *S* is the slope.

DOC was measured on a Shimadzu TOC L_-CSN_ analyzer. Chl-*a* was measured by fluorometry after 90% acetone extraction of the chl-*a* filters. Fe_diss_ and Fe_T_ were analyzed in triplicate with 200 μg/L of yttrium added as an internal standard on a Thermo Scientific iCAP 6500 DUO ICP-OES or iCAP 7600 DUO ICP-OES instrument. Fe_diss_ was not analyzed on some low-CDOM waters sampled in 2016 that, based on 2014–2015 results, were expected to have low Fe_diss_ nor on some high-CDOM samples from lakes sampled multiple times in 2016. Based on analysis of the 2014–2016 data, we collected additional samples in 2018 from some rivers and lakes fed by rivers to measure Fe_T_ and Fe_diss_ and calculated particulate Fe (Fe_part_) by difference.

#### Fe addition experiment

The effect of adding Fe^III^ on *a*_440_ was measured for surface water samples from six northern Minnesota lakes with a range of ambient *a*_440_ and Fe_diss_. The lake waters were circumneutral (pH 6.0–8.0). We used Fe^III^ because Poulin et al. [15] found no effect of Fe^II^ on UV absorbance of CDOM-containing solutions. Fe^III^ is the thermodynamically stable form in oxic water at circumneutral pH, which suggests that Fe^III^-humic complexes predominate in surface waters. A 500 mL aliquot of filtered lake water (0.7 μm glass fiber filters) was placed in a 1.0 L beaker on a magnetic stirrer, and five 0.6 mL increments of a solution containing 77.1 mg/L of Fe^III^ were added sequentially. The additions were designed to yield measurable increases in Fe^III^ (total of 460 μg/L over the five increments) but not over-saturate the DOM. The ratio Fe^III^/DOC was < 1 μmol/mg for the highest additions, lower than reported iron-binding capacities for humic materials, e.g., [21,22]. We also added similar amounts of Fe^III^ to deionized water to determine whether *a*_440_ increased from uncomplexed Fe^III^ and to 0.01 M EDTA to determine whether *a*_440_ increased when Fe^III^ was added to a colorless chelating agent. The Fe^III^ solution was prepared in 0.1 M HNO_3_ from reagent-grade Fe_2_(SO_4_)_3_·nH_2_O, and the resulting Fe^III^ concentration was determined by triplicate ICP-OES analysis. After each Fe^III^ increment, sample pH was adjusted to within 0.1 of its ambient value by dropwise addition of 1 M NaOH. A preliminary experiment showed that the acidic Fe^III^ solution decreased the pH enough to affect the measured *a*_440_. The effect of pH on CDOM absorbance is well known [23]. After pH stabilization, 5 mL aliquots were stored in the dark at 4 ^o^C with no further filtration until absorbance was measured, ~ 24 h later.

#### Data analysis

All observations (site-date combinations) were treated as separate data points; i.e., multiple samples from a lake across or within years were not averaged. Statistical analyses were done in JMP Pro 13.1 except for some simple regressions done in Excel 2016. Initial data inspection showed that distributions for *a*_440_, DOC, and Fe_diss_ were skewed to low values but otherwise well distributed over the range of observed values (S1 Fig). Natural log transforms yielded more Gaussian-looking distributions but still did not satisfy the Shapiro-Wilks test for normality. Unless stated otherwise, statistical results are reported for untransformed data. In addition to simple and multiple regression analyses on subsets of the untransformed and log-transformed data, we analyzed relationships between *a*_440_ and “de-trended” values of DOC and Fe_diss_. The de-trended DOC analysis regressed the residuals from a regression of DOC vs. Fe_diss_ (i.e., the variance in DOC not explained by Fe_diss_) against *a*_440_. The de-trended Fe_diss_ analysis similarly used the residuals from a regression of Fe_diss_ vs. DOC (i.e., the variance in Fe_diss_ not explained by DOC) in a regression vs. *a*_440_.

## Results and discussion

### Overview of water quality variables in study lakes

Broad ranges of *a*_440_, DOC, and Fe_diss_ and two basic limnological variables, SD and chl-*a*, were measured in the study, and large differences were found between the two major ecoregions (NLF and NCHF; Table 1). Median, mean and maximum values of *a*_440_, Fe_diss_, and DOC were substantially higher for NLF lakes (dominated by forests) than NCHF lakes (dominated by agriculture). The median chl-*a* in NLF lakes was 4.1 μg/L (range 0–25 μg/L), and the median in NCHF lakes was 7.6 μg/L (range 1–98 μg/L). Lakes with obvious color (defined here as *a*_440_ > 3.0 m^-1^) also had low chl-*a*, nearly all < 20 μg/L [19]. The SD range was more limited (0.4–5.5 m) in NCHF lakes than NLF lakes (0.3–19.5 m), where high SD values were associated with deep, ultra-oligotrophic mine pit lakes. NLF lakes with *a*_440_ > 3 m^-1^ generally had SD < 3 m, and CDOM levels were the controlling factor for SD in highly colored lakes [24], some of which had SD values as low as 0.3 m. Higher mean than median values for the five variables (especially for *a*_440_ and Fe_diss_) in both ecoregions are indicative of non-normal (skewed) distributions.

**Table 1:** Summary statistics for *a*_440_, DOC, and Fe_diss_ and two basic limnological variables in the NLF and NCHF ecoregions.

	*a*_440_ m^-1^	DOC mg/L	Fe_diss_ μg/L	Chl-*a* μg/L	SD m
**NLF**					
Mean	6.03	12.2	247	4.74	2.3
Standard deviation	7.20	7.8	342	3.54	2.1
Median	2.76	9.2	118	4.06	1.7
Standard error of mean	0.40	0.4	23	0.25	0.1
Skewness	1.6	1.1	2.1	1.9	3.8
Minimum	0	2.5	1	0.02	0.3
Maximum	32.47	36.1	1858	24.9	19.5
Interquartile range	8.33	9.6	346	4.2	1.9
Number of samples	317	313	212	195	234
**NCHF**					
Mean	1.41	8.0	37	16.64	1.7
Standard deviation	1.03	2.7	64	21.49	1.2
Median	1.15	7.7	14	7.56	1.4
Standard error of mean	0.10	0.3	8	2.46	0.2
Skewness	1.40	0.8	3.5	1.90	1.5
Minimum	0.10	3.1	1	1.22	0.4
Maximum	5.30	17.8	391	98.70	5.5
Interquartile range	1.38	3.4	36	15.0	1.5
Number of samples	104	105	64	76	53

**Fig 2:**
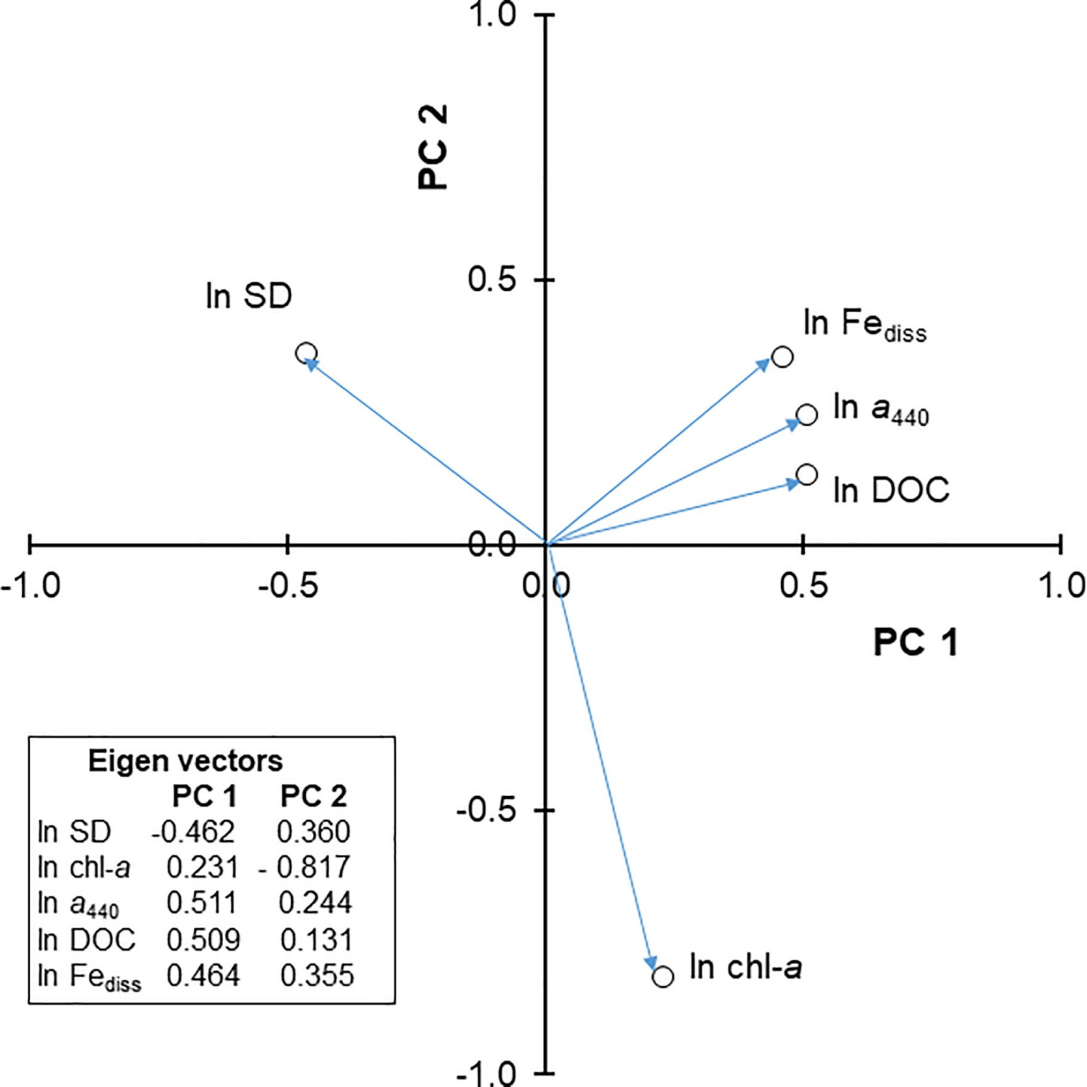
Plot of eigenvalues for the first two principal components of a principal components analysis of five variables (*a*_440_, DOC, Fe_diss_, chl-*a*, and SD) for the whole data set.

A principal components analysis to examine relationships among the above five variables showed that 90% of the variance was explained by the first two principal components (PCs) (Fig 2). DOC, *a*_440_, and Fe_diss_ were clustered together with high positive loadings on PC1, which accounted for 67.2% of the variance. SD also had a high PC1 loading but in a negative direction. PC2, which accounted for 22.5% of the variance, was driven by a high negative loading of chl-*a* and smaller positive loadings of SD and Fe_diss_. Overall, the results support the idea that *a*_440_, Fe_diss_, and DOC behave similarly as variables but behave differently from chl-*a* and SD.

Our 2014–2016 measurements were on near-surface samples because most CDOM effects of interest are near-surface phenomena. Other recent studies on Fe-CDOM interactions, e.g., [3,4], also focused on surface water samples. Data from summer 2018 for lakes with a range of near-surface *a*_440_ indicate that *a*_440_ and Fe_diss_ may vary with depth, with *a*_440_ decreases and Fe_diss_ increases in near-bottom waters of highly colored lakes (S1 Table). These trends could be caused by seasonal variations in CDOM and in-lake cycling processes for Fe and CDOM, a topic beyond the scope of this paper.

All 2014–2016 samples analyzed for Fe were filtered (0.7 μm filters) prior to analysis, and the results are defined operationally as Fe_diss_, which comprises Fe in true solution, including that complexed by DOM, and colloidal Fe associated with macromolecular DOM and hydrous Fe oxide particles too small to be retained on filters. We excluded particulate Fe (Fe_part_) associated with filterable particles from analysis because we considered it inappropriate to include Fe in plankton or mineral particles. Three lines of evidence support this decision.

First, analyses of Fe_T_ and Fe_diss_ on samples collected in 2018 from moderate- to high-CDOM lakes showed that Fe_part_ was only a small fraction of Fe_T_ (average of 10.3%, range 0–23%; S2 Table), and most of Fe_T_ was Fe_diss_. These samples were from lakes with moderate-to-high Fe_diss_ concentrations, and most of the lakes had river inflows. Samples from the associated rivers had a higher fraction of Fe_part_−average of ~ 27%, or ~ 20% when one sample from a high runoff event was excluded (S2 Table). These results agree with the findings of Weyhenmeyer et al. [4], who reported that Fe_part_ was not an important component of Fe_T_ in Swedish lakes. Kritzberg and Ekström [3] found that Fe_part_ was an important fraction of Fe_T_ in Swedish rivers. Our more limited sampling found that Fe_part_ was more important in rivers than lakes, but Fe_diss_ was still dominant in rivers.

Second, concentrations of total suspended matter (TSM) were generally < 10 mg/L in lakes with *a*_440_ > 3.0 m^-1^; the average TSM for 53 lakes sampled in 2016 with *a*_440_ > 3.0 m^-1^ was only 3.8 mg/L. Third, CDOM-rich UGLS lakes occur in highly vegetated, forested catchments, where soil erosion is low, similar in terrain and ecological conditions to the Swedish and Canadian lakes where Fe_part_ was found not to be important [4].

### Fe_diss_ is linearly correlated *a*_440_ and DOC

**Fig 3:**
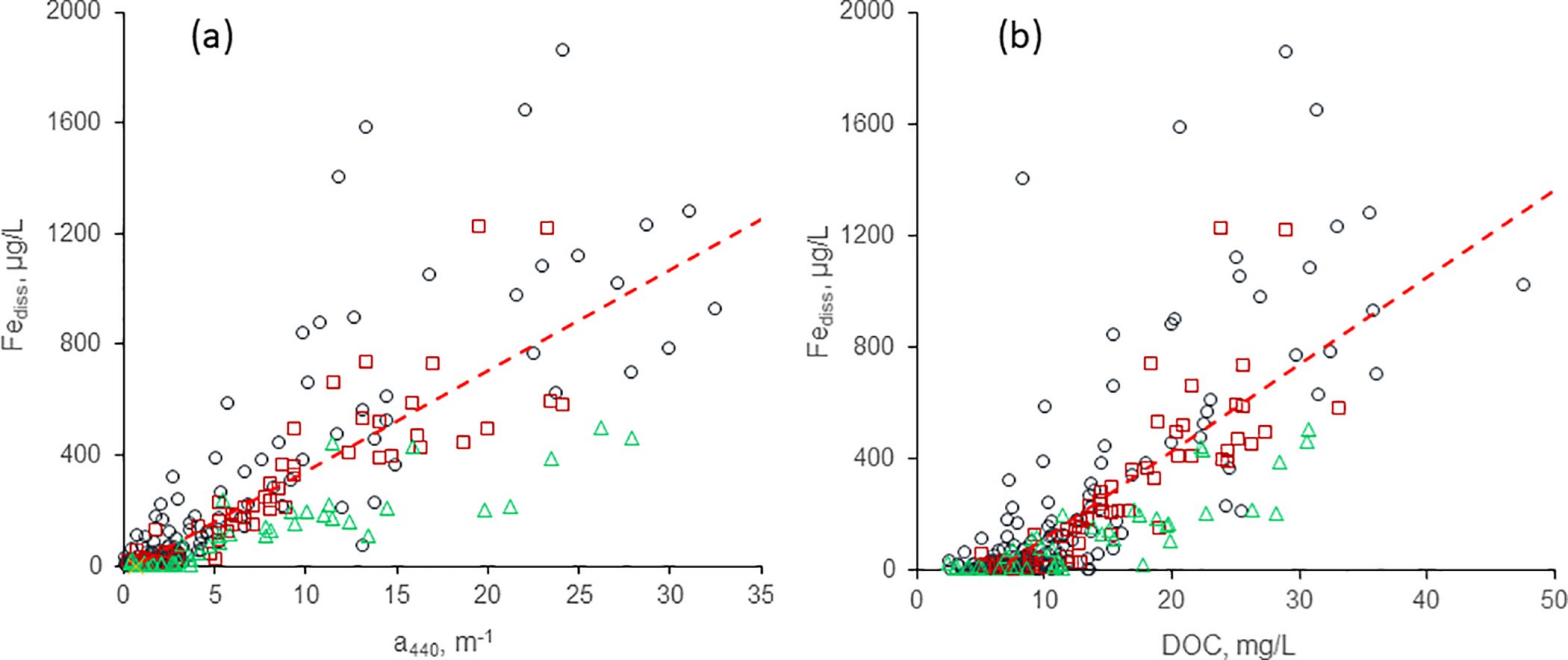
(a) *a*_440_ and (b) DOC vs. Fe_diss_ for sampling years 2104–2016; triangles = 2014, squares = 2015, circles = 2016; dashed lines are regression fits for all years (see Table 1 for regression statistics).

Strong correlations were found between Fe_diss_ and *a*_440_, our measure of CDOM, as well as for Fe_diss_ and DOC, for each year and for the complete data set (Fig 3, Table 2). Similar correlations were obtained for log-transformed data (S3 Table). Values of Fe_diss_ and *a*_440_ generally were higher and more scattered in 2016 than in the two previous years, probably for two reasons. First, unusually high precipitation across Minnesota in 2016 broke many daily and monthly records at individual locations, likely resulted in higher export of Fe and DOM from catchments to lakes, and thus led to higher concentrations. Second, we sampled three times as many sites in 2016 than in 2014 or 2015. These sites covered a larger geographic range and had a greater proportion of catchments in agricultural, urban, or mixed-use landscapes, resulting in a greater diversity of geochemical conditions among sites than for previous years, thus accounting for the greater scatter. Because of the inter-annual differences, R^2^ for the total data set for Fe_diss_ vs. *a*_440_ (0.67) was lower than for the individual years. Overall, however, the results are consistent with century-old [1] and more recent studies [4] associating CDOM and Fe concentrations in lakes.

**Table 2:** Dissolved Fe-CDOM and Fe-DOC relationships.

Year	N	Regression equation^a^	R^2^	RMSE^b^	Slope SE^c^
**Fe**_**diss**_ **(**μ**g/L) vs. *a***_**440**_ **(m**^**-1**^**)**					
2014	46	Fe_diss_ = 17.0×*a*_440_ − 5.8	0.77	66	1.4
2015	61	Fe_diss_ = 37.1×*a*_440_ − 34.3	0.77	131	2.6
2016	175	Fe_diss_ = 43.1×*a*_440_ − 21.9	0.73	184	2.0
All	282	Fe_diss_ = 36.4×*a*_440_ − 23.3	0.67	182	1.5
**Fe**_**diss**_ **(**μ**g/L) vs. DOC (mg/L)**					
2014	42	Fe_diss_ = 14.7×DOC– 76	0.71	76	1.5
2015	61	Fe_diss_ = 32.8×DOC– 241	0.68	154	2.9
2016	177	Fe_diss_ = 36.2×DOC– 218	0.62	218	2.1
All	280	Fe_diss_ = 31.1×DOC– 192	0.58	204	1.6

^a^ All equations and coefficients significant at p < 0.0001.

^b^ Root mean square error

^c^ Standard error of slope.

### DOC is a stronger *a*_440_ predictor than Fe_diss_

Although many catchment and water quality conditions affect lake CDOM levels, we are most interested here in the relative effects of DOC and Fe_diss_ on a_440_ because a_440_ is used to quantify CDOM and often used to predict DOC, e.g., [19]. As shown below, both DOC and Fe_diss_ are strong predictors of *a*_440_, but DOC is stronger. We performed simple and multiple regression analyses with *a*_440_ as predicted variable and DOC and Fe_diss_ as predictor variables (Table 3). Regressions were performed using the entire *a*_440_ range and just for sites with *a*_440_ > 3.0 m^-1^ because related work [19] showed a break in the DOC-*a*_440_ relationship around *a*_440_ = 3.0 m^-1^. A tight fit between the two variables was found above this value, but much more scatter and a higher slope were found below. Griffin et al. [19] interpreted this finding to indicate that low-color DOM from autochthonous and anthropogenic sources was an important, but variable DOC contributor in waters with *a*_440_ < 3.0 m^-1^, and these sources were less important in high-CDOM waters dominated by allochthonous (humic-like) DOM.

**Table 3:** Simple and multiple regression relationships for *a*_440_ vs. DOC and Fe_diss_.

Data range	Best fit equation^a^	N	R^2^	RMSE^b^	SE^c^
All data					
	*a*_440_ = 0.868×DOC − 4.89	434	0.90	2.15	0.015
	*a*_440_ = 0.0183×Fe_diss_ + 2.47	283	0.67	4.08	0.0008
	*a*_440_ = 0.746×DOC + 0.0046×Fe_diss_ − 4.15	277	0.93	1.89	0.023, 0.00056
*a*_440_ > 3.0 m^-1^					
	*a*_440_ = 0.967×DOC − 6.33	159	0.90	2.24	0.025
	*a*_440_ = 0.0141×Fe_diss_ + 5.77	136	0.52	5.10	0.0012
	*a*_440_ = 0.842×DOC + 0.0032×Fe_diss_ − 5.29	134	0.91	2.20	0.035, 0.00068

^a^ Units for variables: *a*_440_ in m^-1^; DOC in mg//L; Fe_diss_ in μg/L. All equations and coefficients significant at *p* < 0.0001.

^b^ Root mean square error.

^c^ SE = standard error for independent variable terms (slopes for simple regressions).

DOC exhibited stronger relationships with *a*_440_ than did Fe_diss_, but both variables were significant in multiple regressions (Table 3). Addition of Fe_diss_ as a second variable increased R^2^ by only 0.03 for the entire *a*_440_ data range and 0.01 for *a*_440_ > 3.0 m^-1^. Similar results were found using log-transformed data (S4 Table), except that R^2^ increased more when adding Fe_diss_ as a second variable (0.09 for all data, 0.03 for *a*_440_ > 3.0 m^-1^). De-trending to remove the influence of Fe_diss_ on the *a*_440_–DOC relationship yielded an R^2^ of 0.32. Removal of the influence of DOC on the *a*_440_-Fe_diss_ relationship yielded an even lower R^2^ of 0.12.

Weyhenmeyer et al. [14] similarly found that a least squares model using ln DOC and ln Fe_diss_ explained 86% of the variance in ln *a*_420_. Linear de-trending of their data showed that DOC explained 38% of the variance when the Fe signal had been removed, and Fe explained 25% of the variance when the DOC signal was removed. Comparable de-trended values for ln-ln relationships of our data are 25% for DOC with the Fe_diss_ signal removed and 12% for Fe_diss_ when the DOC signal was removed. Although numerical values of Weyhenmeyer et al.’s original and de-trended R^2^ results differ from ours, the overall outcomes of the analyses are similar: de-trending caused a large decrease in fit for *a*_λ_-DOC relationships and even a larger decrease for *a*_λ_-Fe_diss_ relationships. Together, these findings indicate that DOC is the more important explanatory variable statistically, but Fe_diss_ does explain some variance in *a*_440_ beyond that produced by the correlation between DOC and Fe_diss_.

As noted above, DOC and Fe_diss_, the two main chemical determinants of *a*_440_, are themselves moderately correlated for the complete data set (Fig 3B) and within each year. In each case, R^2^ for the Fe_diss_-DOC relationship was lower than that for the corresponding Fe_diss_-*a*_440_ relationship (Table 2), and 2016 values were more scattered than those for the previous years; R^2^ for the total data set was only 0.58. Regression equations between Fe_diss_ and *a*_440_ (Table 2, Fig 3A) had x-intercepts of *a*_440_ < 1 m^-1^. In contrast, best-fit lines for linear regressions of Fe_diss_ vs. DOC had x-intercepts of 5–7 mg/L DOC (Table 2, Fig 3B). Together, these findings suggest that (i) Fe_diss_ is associated with the colored component of DOM and (ii) on average across all sites ~ 6 mg/L of DOC is not associated with Fe_diss_. This likely represents low-color DOM with a low abundance of Fe-binding ligand groups, probably of autochthonous or anthropogenic origin. Photo-degradation of CDOM also could contribute to the low-color DOM pool, but it is uncertain whether CDOM photo-degradation reduces Fe binding capacity.

**Fig 4:**
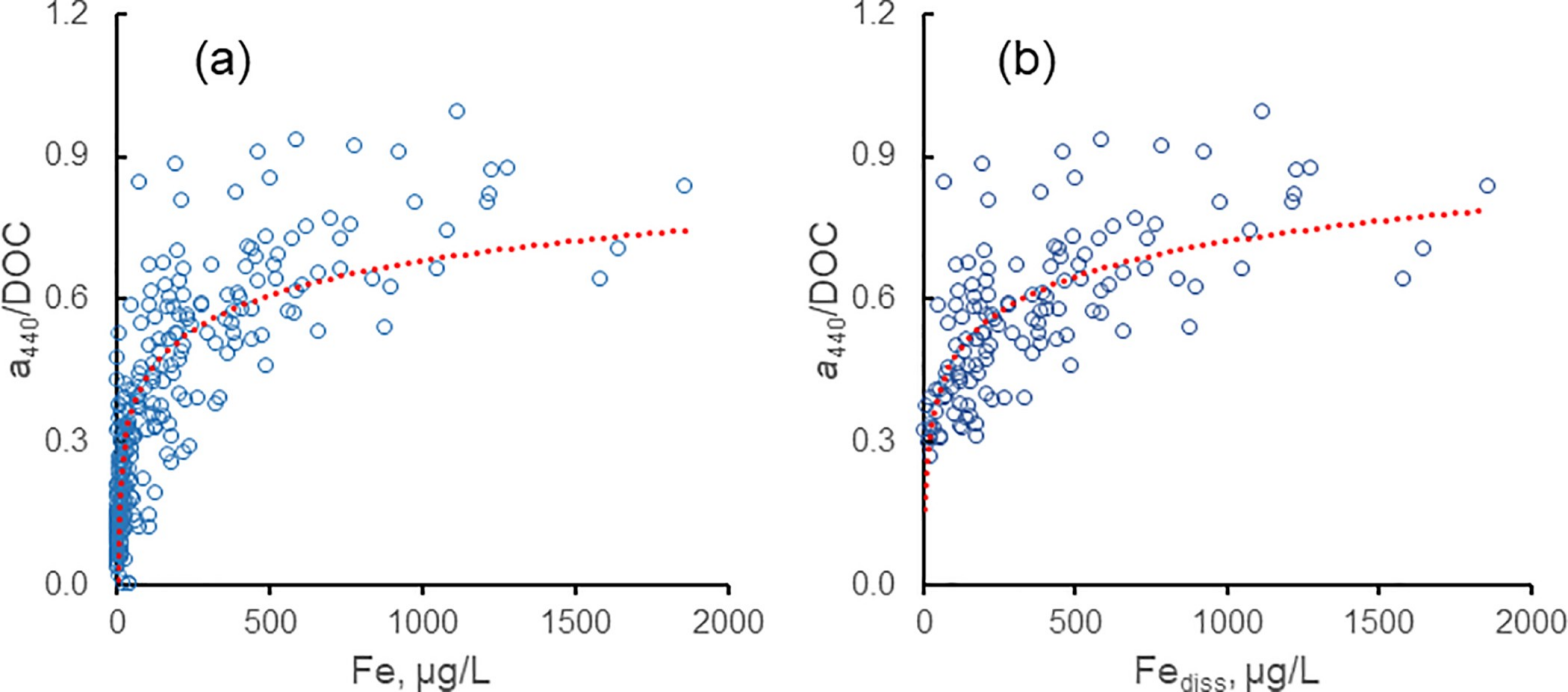
(a) *a*_440_/DOC vs. Fe_diss_ for all data, R^2^ = 0.64; (b) *a*_440_/DOC vs. Fe_diss_ for sites with *a*_440_ > 3.0 m^-1^, R^2^ = 0.46. (R^2^ are for fit of *a*_440_/DOC to ln(Fe_diss_)).

Weyhenmeyer et al. [14] found a curvilinear relationship (R^2^ = 0.49) between the ratio *a*_λ_/DOC and Fe_diss_ that might be interpreted as a measure of the effect of Fe_diss_ on the fraction of DOC that is colored. We found a similar relationship (Fig 4A) for our data; R^2^ = 0.64 for *a*_440_/DOC vs. ln Fe_diss_. As discussed above, however, the nature of DOM in low-CDOM waters (*a*_440_ < 3.0 m^-1^) likely differs from that in high-CDOM waters. The latter consists primarily of allochthonous, humic-like DOM; the former derives from various sources with generally lower color intensity and probably fewer binding sites for Fe_diss_. Consequently, trends in *a*_440_/DOC vs. Fe_diss_ may simply reflect changes in the nature of DOM as *a*_440_/DOC increases. A plot of the relationship for sites dominated by allochthonous DOM (those with *a*_440_ > 3.0 m^-1^; Fig 4B) yielded an R^2^ of only 0.46, and there was little trend in the ratio for Fe_diss_ > 300 μg/L. Overall, the close fit between *a*_440_ and DOC for waters with *a*_440_ > 3.0 m^-1^ (Table 3) suggests that the DOM for these sites was dominated by humic-colored DOM.

### Fe_diss_ had minor effects on other CDOM optical properties

**Fig 5:**
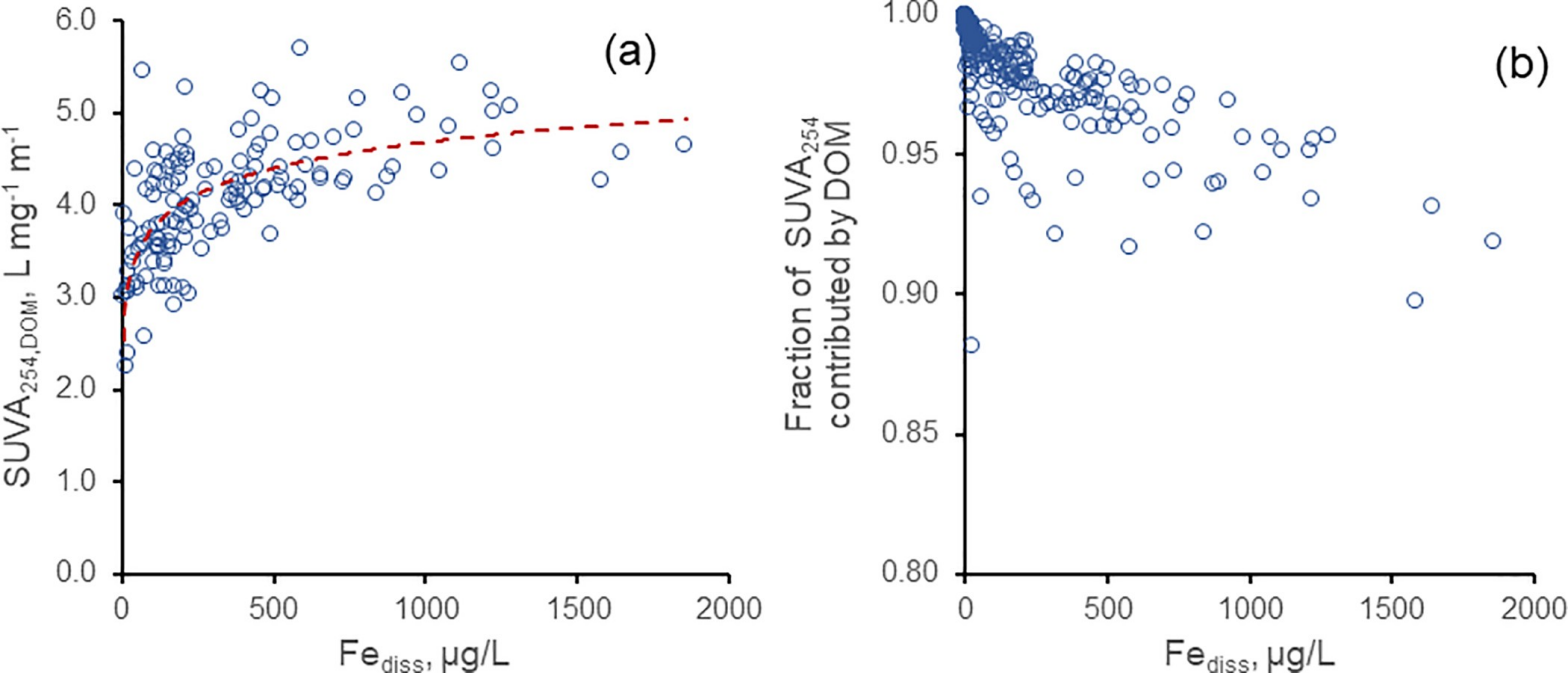
(a) SUVA_254,DOM_ vs. Fe_diss_ for sites with *a*_440_ > 3.0 m^-1^, R^2^ = 0.45 (R^2^ is for fit to ln(Fe_diss_)); (b) fraction of SUVA_254_ for all sites caused by DOM (i.e., corrected for Fe^III^ contribution) vs. Fe_diss_.

The above results show that Fe_diss_ should be considered when evaluating *a*_440_. Thus, it is worthwhile to assess whether other common optical measurements also are affected by Fe_diss._ Results similar to those for *a*_440_/DOC were obtained for SUVA_254_, a more common DOC-normalized optical measure. For the whole data set, a moderate fit was found for both uncorrected SUVA_254_ vs. ln Fe_diss_ (R^2^ = 0.67) and for SUVA_254,DOM_ vs. ln Fe_diss_ (R^2^ = 0.64). For samples dominated by allochthonous DOM (*a*_440_ > 3.0 m^-1^), both relationships had lower R^2^ (0.50 and 0.45, respectively, for uncorrected and Fe-corrected SUVA_254_), with little trend above Fe_diss_ = 300 μg/L (Fig 5A).

Fe_diss_ contributions to SUVA_254_, calculated according to [15], were small (mean = 1.9%, std. dev. = 1.9%, n = 271); on average, across all UGLS samples with Fe_diss_ and SUVA_254_ data, more than 98% of the SUVA_254_ signal thus could be attributed to DOM. A small number of samples, however, had larger Fe_diss_ contributions to SUVA_254_ (Fig 5B); 18 had Fe_diss_ contributions > 5%, and two had contributions > 10%. The lake with the largest contribution (11.9%), Crystal Lake (WI), is an ultra-clear oligotrophic seepage lake with low DOC (2.5 mg/L) and a SUVA_254_ of only 0.64 L mg^-1^ m^-1^; it is an outlier relative to most lakes in the region. More relevant here are samples with higher Fe_diss_ and DOC. Seven samples with Fe_diss_ of 1000–1500 μg/L, had Fe_diss_ contributions to SUVA_254_ of 4.4–6.7%, and Fe_diss_ contributions for three samples with Fe_diss_ > 1500 μg/L were 6.9–10.3%.

SUVA_254_ values corrected for Fe_diss_ were slightly lower than uncorrected values, but of the 15 samples with original SUVA_254_ > 5.0 L mg^-1^ m^-1^, 10 still had values > 5.0 after correction. The common upper limit for DOM-caused SUVA in natural waters is 5.0 L mg^-1^ m^-1^ [15]. Average SUVA_254_ values for the 15 samples before and after correction (S5 Table) were 5.33 and 5.15 L mg^-1^ m^-1^, respectively. A third of these samples were from Johnson Lake, Minnesota (Itasca County), a small bog lake that generally had the highest CDOM and SUVA_254_ levels in our studies. The average SUVA_254_ before Fe-correction for Johnson Lake of 5.41 L mg^-1^ m^-1^ decreased to 5.23 L mg^-1^ m^-1^ after correction. Although high nitrate/nitrite concentrations (tens of mg/L range) may affect levels of SUVA_254_ [25], concentrations of these ions were very low (few μg/L) in Johnson Lake and the other lakes we studied.

Spectral slopes, a measure of DOM composition, are also influenced by Fe_diss_ [16,17]. Plots of the spectral slopes *S*_350-400_ and *S*_400-460_ versus the ratio Fe_diss_/*a*_440_ showed no trends, but *S*_275-295_ had a trend of smaller slopes with increasing Fe_diss_/*a*_440_, albeit with considerable scatter. *S*_275-295_ values > 0.020 generally were from sites with *a*_440_ < 3.0 m^-1^, where low-colored autochthonous and anthropogenic DOM was dominant. Sites dominated by allochthonous DOM (*a*_440_ > 3.0 m^-1^) had lower scatter, but the trend explained little variance in *S*_275-295_ (R^2^ = 0.13). The trend in *S*_275-295_ generally agrees with findings of others [16,17], who reported that Fe_diss_ decreased spectral slopes. The lack of trends in *S*_350-400_ and *S*_400-460_, however, reinforces the conclusion that Fe_diss_ at levels found in UGLS lakes does not strongly influence absorbance in the UV-A and visible regions.

### Addition of Fe_diss_ had minor effects on *a*_440_

**Fig 6:**
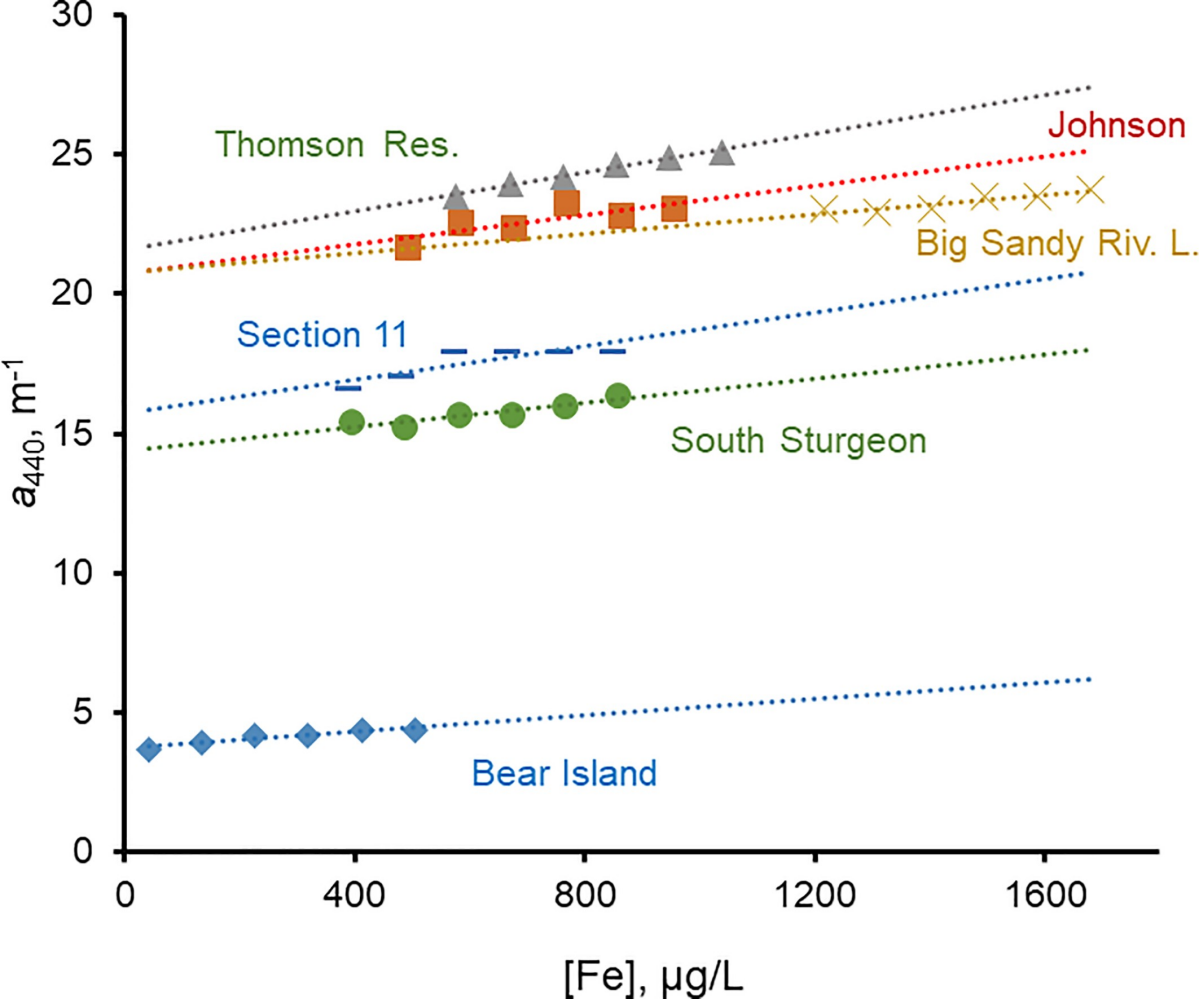
*a*_440_ vs. Fe_diss_ for six waters in iron addition experiment; slopes and statistical information on best-fit lines in Table 4.

To measure effects of Fe_diss_ on *a*_440_ directly, we added known amounts of an acidified Fe^III^ solution to six lake waters with a range of *a*_440_, DOC, and Fe_diss_ (Table 4). Although *a*_440_ increased linearly with added Fe^III^ after readjusting the pH to the original value (Fig 6), the rate was small. The average rate of increase, 0.242 m^-1^ per 100 μg/L of added Fe^III^, was within the range observed by others: 0.19 and 0.29 m^-1^ per 100 μg/L of added Fe^III^ ([3,17], respectively). The weak response to Fe^III^ additions indicates that changes in Fe_diss_ have only small effects on *a*_440_, and inspection of absorbance spectra over the range 250–500 nm showed no changes in shapes of the spectra. Addition of 3.0 mL of acidified stock Fe^III^ solution to 500 mL of deionized water or to 500 mL of 0.01 M EDTA at pH 6.5 yielded no measurable increases in *a*_440_.

**Table 4:** Chemical characteristics of lakes and results for iron addition experiment.

Lake	*a*_440_ m^-1^	*a*_254_ m^-1^	DOC mg/L	SUVA L m^2^/g	Fe_diss_ μg/L	Fe_diss_/DOC μg/mg	Slope, *a*_440_ (m^-1^) per 100 μg/L of added Fe_diss_	R^*2*^, *a*_440_ vs. added Fe_diss_
Bear Island	3.7	87	12.7	3.4	42	3.3	0.15	0.92
So. Sturgeon	15.4	253	24.0	4.6	396	16.5	0.22	0.84
Section 11	16.6	256	24.4	4.5	387	15.9	0.30	0.71
Johnson	21.6	310	27.4	4.9	492	17.9	0.26	0.63
Big Sandy River Lake	23.0	354	29.0	5.3	1217	41.9	0.17	0.84
Thomson Reservoir	23.5	363	33.2	4.7	577	17.3	0.35	0.98

If the relationships in Fig 6 apply across the entire Fe^III^ range, a substantial fraction of the ambient *a*_440_ remains at Fe_diss_ = 0; extrapolating the best-fit lines in Fig 6 to the ordinate yielded *a*_440_ values in the range 90.3–99.7% (mean of 95.5%) of the ambient *a*_440_. On average for the six lakes, ~ 95% of measured *a*_440_ thus can be attributed to DOM and only ~ 5% to enhanced absorptivity from Fe_diss_ or Fe_diss_-DOM complexes. Xiao et al. [17] reported that Fe_diss_ (or Fe-DOM complexes) was responsible for up to 56% of *a*_410_ in 13 natural waters. Their highest value, however, was from a Finnish groundwater spring with very low *a*_410_ (0.2 m^-1^) and Fe_diss_ = 42 μg/L. DOC was not reported but likely very low. It was an outlier among the waters, and Fe contributions to *a*_410_ for the 12 other samples were 0.6–8.7% (mean = 2.8%).

The fact that *a*_440_ did not increase when Fe^III^ was added to DI water or to an EDTA solution but increased by small amounts when added to CDOM-rich waters indicates that the increase is caused by interaction of Fe^III^ with DOM molecules and not Fe^III^ absorbance itself. The chemical nature of Fe interactions with DOM is complicated [26], and how they may affect absorption of visible light (e.g., at 440 nm) still is not well understood. Fe^III^-complexes with carboxylate groups in humic substances can undergo photochemical reduction to Fe^II^ [27,28], and the occurrence of Fe^II^-humic complexes in oxic waters thus cannot be ruled out. There also is evidence that some Fe associated with aquatic humic substances is bound irreversibly, apparently not as conventional metal-ligand complexes [29–31]. The literature has conflicting information on Fe^II^ stability in the presence of humic substances. Complexation by humic substances inhibited Fe^II^ autoxidation rates (oxidation by O_2_) [32], but fulvic acid accelerated Fe^II^ oxidation by hydrogen peroxide (an intermediate in O_2_ reduction to H_2_O) [33]. Nonetheless, several studies [34,35] reported that Fe^III^ forms stronger complexes with DOM than Fe^II^ and probably is the predominant Fe-DOM form in oxic waters. Stability constants (*K*_f_) for Fe^III^ and Fe^II^ with 12 DOM sources [35] were 10^2^−10^4^ higher for Fe^III^ than for Fe^II^ although stability constants varied widely among the DOM sources. Overall, our results indicate that Fe^III^ complexation by DOM has very small effects on CDOM chromophoric groups.

### Application of experimental results to field data

**Fig 7:**
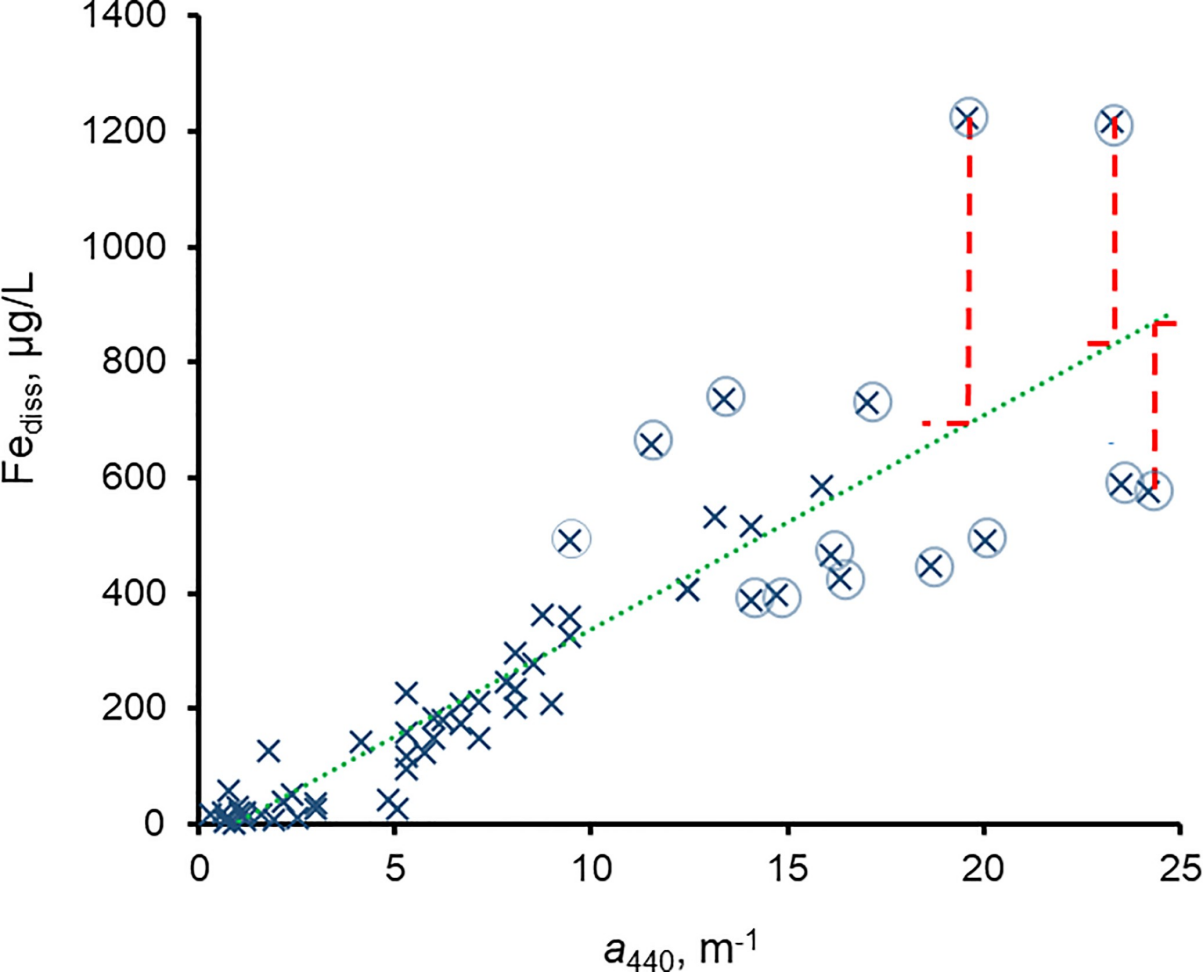
Fe_diss_ vs. *a*_440_ for 2015 data; best-fit regression line and statistics given in Table 2. Circled data: outliers examined for effects of Fe_diss_ on *a*_440_ (S6 Table). Dotted line: best-fit linear relationship: Fe_diss_ = 36.8*a*_440_−30.7; R^2^ = 0.79. Dashed lines illustrate change in *a*_440_ for some outliers when Fe_diss_ was changed to “best-fit” value.

We applied the experimental results to our field data to further evaluate Fe_diss_ effects on *a*_440_, which is critical to know before attempting to use *a*_440_ to predict DOC. For example, the Fe_diss_-*a*_440_ regression of the 2015 data (Fig 7) showed that some data points were far from the regression line. For the largest outliers (six high and eight low), we estimated the change in *a*_440_ that would occur if Fe_diss_ were adjusted to the “best fit” values of the regression relationship. The difference between measured and best-fit Fe_diss_, multiplied by 0.242 m^-1^ per 100 μg/L of Fe_diss_ (average slope of the *a*_440_-Fe^III^ relationship, Fig 6), provided estimates of the *a*_440_ change caused by the Fe_diss_ change. The results showed small *a*_440_ changes even for waters with large differences between measured and best-fit Fe_diss_. For example, Blueberry Lake had the highest measured Fe_diss_ (1224 μg/L), and the best-fit Fe_diss_ for its measured *a*_440_ (19.6 m^-1^) is 690 μg/L. If the latter value represented the Fe_diss_ in this lake, *a*_440_ would be 18.3 m^-1^, a decrease of 1.3 m^-1^ (a 6.6% change). Similar changes were found for the other waters with large differences between measured and best-fit Fe_diss_ (S6 Table); the average *a*_440_ change for the six high outliers was– 3.7% (range −1.8 to − 6.6%), and the average for the eight low outliers was +2.2% (range 1.2 to 2.9%).

“Iron-corrected” *a*_440_ values for samples with Fe_diss_ data are estimates of the *a*_440_ attributable to DOM alone (*a*_440,OM_). These were obtained by multiplying measured Fe_diss_ values by 0.242 (average slope of the *a*_440_-Fe^III^ relationships in Fig 6) and subtracting the result from measured *a*_440_. For 136 waters with *a*_440_ > 3.0 m^-1^, *a*_440,OM_ was 92.3 ± 5.0% (range of 71.3–99.7%) of measured *a*_440_. Fe_diss_ accounted for < 10% of *a*_440_ in most lakes (102, or 75%), but in seven lakes it accounted for 15–30% of measured *a*_440_ (S7 Table). These lakes generally were river-influenced systems with relatively high Fe_diss_ concentrations and/or high Fe_diss_/DOC ratios, and all were samples collected in the high rainfall year 2016. Of the six lakes that also had data for 2014 or 2015, only Big Sandy Lake and Big Sandy River Lake had Fe_diss_ contributions to *a*_440_ > 10% in those years. We conclude that high rainfall promotes Fe export to lakes, resulting in higher Fe_diss_ contributions to *a*_440_, and that lakes influenced by rivers with high CDOM and Fe are more likely to have relatively high Fe_diss_ contributions to *a*_440_.

### DOC can be predicted from a_440_ without correcting for Fe_diss_

It is now possible to assess whether correction for the presence of Fe_diss_ is needed to allow accurate prediction of DOC from *a*_440_. Regressions of measured DOC versus *a*_440,OM_ and measured DOC versus measured *a*_440_ yielded very similar relationships for the same data set (*a*_440_ > 3.0 m^-1^; N = 134):

$$\ln\left({\mathrm{DOC}}_{meas}\right)=0.587\times \ln\left(a_{440,\mathrm{OM}}\right)+1.57;\;R^{2}=0.86,RMSE=0.146,slope\;SE=0.020,p<0.0001$$ (3)

$$\ln\left({\mathrm{DOC}}_{meas}\right)=0.573\times \ln\left(a_{440,meas}\right)+1.55;\;R^{2}=0.85,\mathrm{RMSE}=0.154,\mathrm{slope}\;SE=0.020,p<0.0001$$ (4)

The DOC predicted by Eq 3 from the estimated *a*_440,OM_ for each site was compared to the DOC predicted from the measured *a*_440_-DOC relationship (Eq 4); the relationship was almost exactly 1:1 (slope = 0.997; R^2^ = 0.99) (S2 Fig). Consequently, we conclude that DOC predictions from measured *a*_440_ are just as accurate for our study sites as DOC predictions from *a*_440_ corrected to remove the influence of Fe_diss_. Moreover, for waters where *a*_440_ > 3.0 m^-1^, *a*_440_ is a good predictor of DOC (Table 3).

### Long-term trends in CDOM and the role of Fe_diss_ in UGLS lakes

Long-term data across UGLS surface waters for CDOM [18] and Fe_diss_ are scarce. The extent of regional increases in CDOM, or whether such increases could be attributed to increases in Fe_diss_, thus is unknown. Smaller-scale analyses suggest, however, that regional CDOM trends are more complicated than observed in Scandinavia and likely driven by climatic and hydrologic variations. For example, substantial intra- and inter-annual variations but no monotonic trends were found in *a*_440_ and DOC for 20 small lakes in Upper Michigan over six years [36]. Climatic conditions that affected carbon loadings from upland forests and wetlands were considered the drivers of these variations. Similarly, Brezonik et al. [18] found large *a*_440_ variations in seven lakes of the northern Wisconsin LTER program (all within a radius of 10 km, [37]) for the period 1990–2012, but only colored Crystal Bog had increasing *a*_440_ over the whole period. In a study on optical properties of the LTER lakes, Jane et al. [37] found inconsistent trends in DOC since 1990, with increases in two (including Crystal Bog), decreases in four, and no trend in one. Optical properties related to DOM chemical characteristics varied more with climatic conditions than DOC concentrations.

Björnerås et al. [38] recently reported temporal increases in Fe on broad scales in European and North American freshwaters. Their data overlap our region at only one site (in north-central Wisconsin). Additional data from the Wisconsin LTER lakes, which were not included in the Björnerås et al. study, showed that Fe increased in Crystal Bog, decreased in Trout Bog, and had no trends in the other five lakes since the 1980s (Mann-Kendall test; N. Lottig, Univ. Wisconsin, pers. comm., 2017). As noted above, Crystal Bog is the only LTER lake with increasing CDOM during the same period. The intra- and inter-annual variability in Fe and CDOM was high in all the lakes. In Crystal Bog, Fe_diss_ averaged 200 μg/L for six pre-1990 measurements and 330 μg/L for eight post-2010 measurements; the average increase of 130 μg/L could account for an *a*_440_ increase of only ~ 0.3 m^-1^ (based on the average slope in Fig 6), but *a*_440_ in Crystal Bog actually increased by ~ 5.5 m^-1^ over this time [18]. Moreover, pH data for Crystal Bog showed no trends over the period of record (1981–2016) (S3 Fig). The increases in DOC [37] and *a*_440_ [18] in Crystal Bog thus cannot be explained by declining acidity, and the lack of similar trends in the other LTER lakes suggests that long-term climatic changes also are not responsible for the trends.

## Conclusions

Our examination of the role of dissolved iron in optical properties of DOM in lakes supports three main conclusions. First, *a*_440_ and Fe_diss_ are well correlated in surface waters of the UGLS, with R^2^ values of 0.73–0.77 for individual years, as has been shown in studies elsewhere. Second, experimental data show that iron has small effects on CDOM measured as *a*_440_, but it is not the dominant factor for *a*_440_ or SUVA_254_ variations in UGLS lakes. The average increase in *a*_440_ with added Fe_diss_ (0.242 m^-1^ per 100 μg/L) means that increasing Fe_diss_ by 400 μg/L would increase *a*_440_ by only ~ 1.0 m^-1^. Even this level of Fe_diss_ variation leads to an *a*_440_ change less than the expected error in using *a*_440_ as a proxy for DOC (RMSE of 1.3–1.8 m^-1^, Table 3). Third, estimates of DOC based on measured *a*_440_ and *a*_440,DOM_ (i.e., *a*_440_ corrected for Fe_diss_) were essentially the same. Consequently, our data indicate that ambient levels of Fe_diss_ have only a minor influence on CDOM optical properties (*a*_440_ and SUVA_254_) and do not affect DOC estimates based on *a*_440_ in lakes of our study area.

## Supporting information

S1 Fig Histograms of data distributions for *a*_440_ (CDOM), DOC, Fe_diss_, and SUVA_254_. Upper plots: untransformed data; lower plots: log-transformed (ln) values.
(DOCX)

S2 Fig DOC predicted from Fe-corrected *a*_440_ (*a*_440,OM_) (Eq 3) vs. DOC predicted from measured *a*_440_. (Eq 4). Best-fit line: *a*_440,OM_ = 0.997*a*_440_ + 0.032; R^2^ = 0.99; RMSE = 0.685, slope SE = 0.0086, *p* < 0.0001.
(DOCX)

S3 Fig Time trend for pH in Crystal Bog, Vilas County, Wisconsin, 1981–2016; data from the North Temperate Lakes Long Term Ecological Research (LTER) program (http://lter.limnology.wisc.edu). (DOCX)

S1 Table Vertical profile data for three NLF lakes with a wide range of surface CDOM (*a*_440_) values. (DOCX)

S2 Table Fe_T_, Fe_diss_, and % Fe_diss_ for 2018 lake and associated river samples from the NLF ecoregion. (DOCX)

S3 Table Fe_diss_-*a*_440_ and Fe_diss_-DOC relationships for log-transformed data. (DOCX)

S4 Table Log-transformed regression relationships for *a*_440_ vs. DOC and Fe_diss_. (DOCX)

S5 Table SUVA_254_ values for samples with measured SUVA_254_ > 5.0 before and after Fe_diss_ correction. (DOCX)

S6 Table Changes in *a*_440_ for 2015 waters that had large differences between measured and best-fit Fe_diss_ after Fe_diss_ was changed to the best-fit value. (DOCX)

S7 Table Samples with measured *a*_440_ > 3.0 m^-1^ having 15–30% of *a*_440_ caused by Fe_diss_ and 70–85% caused by colored DOM. (DOCX)
