# Supplementary material for: Iron influence on dissolved color in lakes of the Upper Great Lakes States
Source: PLoS One. 2019 Feb 13;14(2):e0211979. doi: 10.1371/journal.pone.0211979 (PMC6373958; doi:10.1371/journal.pone.0211979)
Supplement: S1 Fig — Upper plots: untransformed data; lower plots: log-transformed (ln) values. (DOCX) [file pone.0211979.s001.docx]

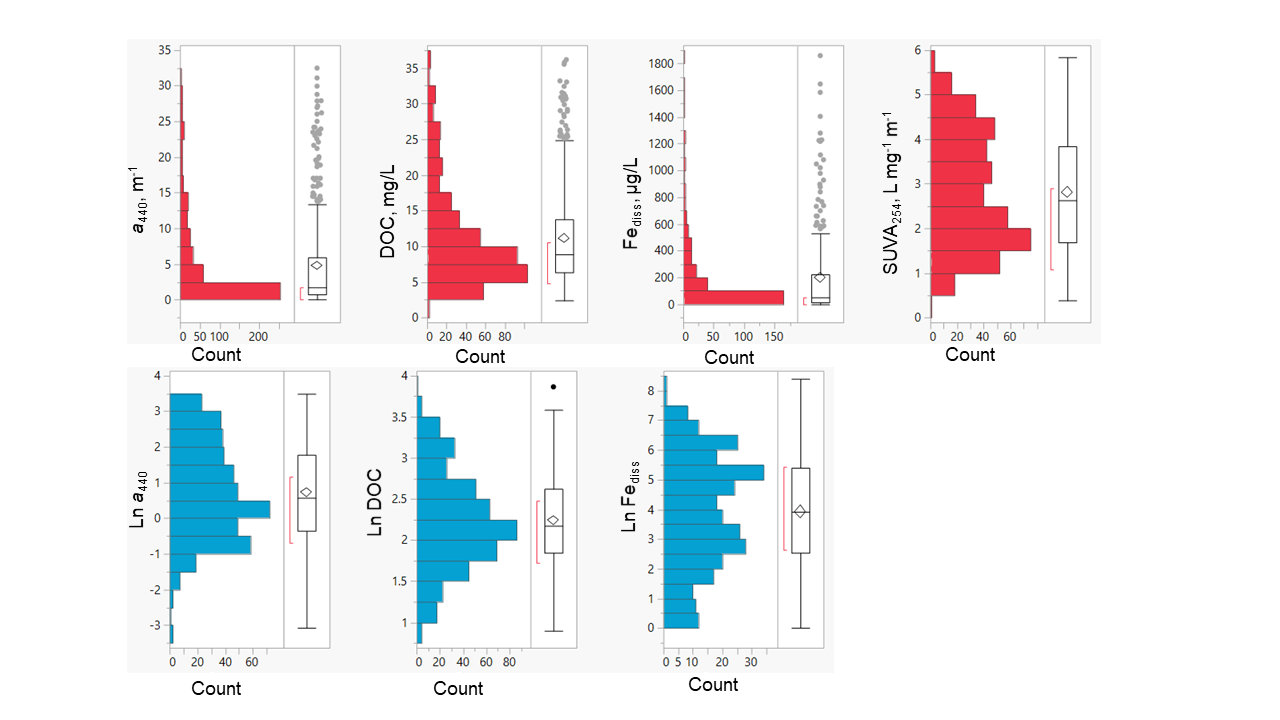


**Fig S1. Histograms of data distributions for *a*_440_ (CDOM), DOC, Fe_diss_, and SUVA_254_. Upper plots: untransformed data; lower plots: log-transformed (ln) values.**
