# Supplementary material for: Iron influence on dissolved color in lakes of the Upper Great Lakes States
Source: PLoS One. 2019 Feb 13;14(2):e0211979. doi: 10.1371/journal.pone.0211979 (PMC6373958; doi:10.1371/journal.pone.0211979)
Supplement: S2 Fig — (Eq 4). Best-fit line: a440,OM = 0.997a440 + 0.032; R2 = 0.99; RMSE = 0.685, slope SE = 0.0086, p < 0.0001. (DOCX) [file pone.0211979.s002.docx]

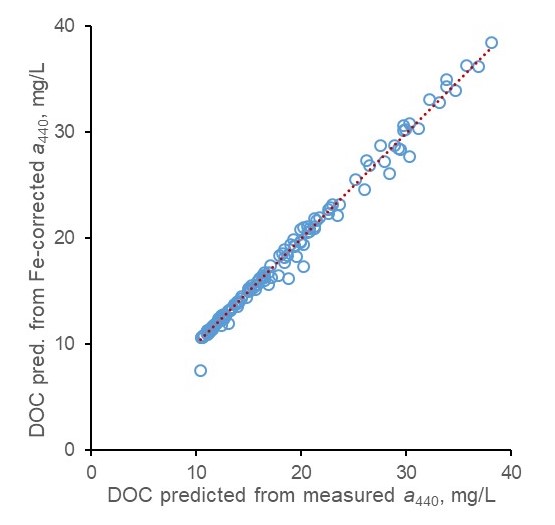


**Fig S2. DOC predicted from Fe-corrected *a*_440_ (*a*_440,OM_) (equation 3) vs. DOC predicted from measured *a*_440_. (equation 4). Best-fit line: *a*_440,OM_ = 0.997*a*_440_ + 0.032; R^2^ = 0.99; RMSE = 0.685, slope SE = 0.0086, *p* < 0.0001.**
