# Supplementary material for: Iron influence on dissolved color in lakes of the Upper Great Lakes States
Source: PLoS One. 2019 Feb 13;14(2):e0211979. doi: 10.1371/journal.pone.0211979 (PMC6373958; doi:10.1371/journal.pone.0211979)
Supplement: S3 Fig — (DOCX) [file pone.0211979.s003.docx]

**Fig S3. Time trend for pH in Crystal Bog, Vilas County, Wisconsin, 1981-2016; data from the North Temperate Lakes Long Term Ecological Research (LTER) Program (**[**http://lter.limnology.wisc.edu**](http://lter.limnology.wisc.edu)**).** The early part of the period of record (1981-1983) shows an apparent pH decline followed by a period of increasing pH (~1984-1987) and another period of decline (~1988-1995). The whole 35-year period, however, has no significant pH trend. Regression of pH vs. year for the entire period yielded a very small, non-significant slope: pH = 5.11 + 0.0011(Year); Adj. R^2^ = 0.0010; RMSE = 0.26; N = 1175; *p* = 0.138 (NS) for slope. Jane et al. (2017) reported a very small, non-significant, declining trend in Crystal Bog pH using the non-parametric Sen’s slope test on seasonally de-trended pH data.
