# Supplementary material for: Iron influence on dissolved color in lakes of the Upper Great Lakes States
Source: PLoS One. 2019 Feb 13;14(2):e0211979. doi: 10.1371/journal.pone.0211979 (PMC6373958; doi:10.1371/journal.pone.0211979)
Supplement: S1 Table — (DOCX) [file pone.0211979.s004.docx]

**S1 Table. Vertical profile data for three NLF lakes with a wide range of surface CDOM (*a*_440_) values.^1^**

| **Lake name** | **Depth m** | **T °C** | **PAR μM m^-2^ s^-1^** | **SC μS/cm** | **pH n** | ***a*_440_ m^-1^** | **Fe_diss_  μg/L** |
| --- | --- | --- | --- | --- | --- | --- | --- |
| **Sturgeon** |  |  |  |  |  |  |  |
|  | 0 | 26.9 | 293.8 | 76.7 | 8.22 | 1.3 | 22 |
|  | 2 | 24.6 | 54.9 | 76.2 | 8.24 | 1.3 |  |
|  | 4 | 23.6 | 25.2 | 75.9 | 7.80 | 1.6 |  |
|  | 6 | 18.4 | 5.9 | 77.7 | 7.84 | 1.2 |  |
|  | 8 | 11.4 | 1.3 | 78.0 | 7.10 | 1.0 |  |
|  | 10 | 8.8 | 0.5 | 77.8 | 6.82 | 0.9 |  |
|  | 16 |  |  |  |  | 0.7 | 26 |
| **West Sturgeon** | |  |  |  |  |  |  |
|  | 0 | 28.2 | 156 | 62.1 | 7.40 | 19.1 | 775 |
|  | 2 | 23.5 | 0.04 | 62.5 | 7.13 | 21.0 |  |
|  | 4 | 12.5 |  | 71.7 | 6.99 | 13.3 |  |
|  | 6 | 7.9 |  | 80.3 | 6.40 | 9.8 |  |
|  | 8 | 6.4 |  | 83.7 | 6.22 | 11.9 | 1928 |
| **South Sturgeon** | |  |  |  |  |  |  |
|  | 0 | 24.0 | 54.7 | 53.7 | 6.58 | 29.2 | 1278 |
|  | 2 | 23.8 | 0.0 | 54.2 | 6.53 | 29.9 |  |
|  | 4 | 12.7 |  | 57.2 | 6.49 | 19.2 |  |
|  | 6 | 7.7 |  | 69.4 | 6.13 | 15.0 |  |
|  | 8 | 6.7 |  | 72.0 | 5.93 | 15.2 | 1571 |

**^1^ Notes:**

1. Lakes were sampled on July 10-11, 2018 and are located near Side Lake, MN in western St. Louis County.
2. Temperature, specific conductance (SC), and pH were measured in situ by a YSI Exo2 water quality sonde with a 10-m cable. Photosynthetically active radiation (PAR) was measured with Li-cor LI-250A light meter, and *a*_440_ and Fe_diss_ were measured in the laboratory on samples collected at depth by a Van Dorn sampler using methods described in the text.
3. All three lakes were thermally stratified on the measurement date. Note the increase in SC and decrease in pH in water below the thermocline.
4. Note the very rapid decrease in PAR with depth in highly colored West and South Sturgeon Lakes compared with the less rapid decline in low-CDOM Sturgeon Lake.
