# Supplementary material for: Iron influence on dissolved color in lakes of the Upper Great Lakes States
Source: PLoS One. 2019 Feb 13;14(2):e0211979. doi: 10.1371/journal.pone.0211979 (PMC6373958; doi:10.1371/journal.pone.0211979)
Supplement: S2 Table — (DOCX) [file pone.0211979.s005.docx]

**S2 Table. Fe_T_, Fe_diss_, and % Fe_diss_ for 2018 lake and associated river samples from the NLF ecoregion.**

|  | **Sampling Date** | **Fe_diss_ μg/L** | **Fe_T_ μg/L** | **% Fe_diss_** |
| --- | --- | --- | --- | --- |
| **(A) individual sites** |  |  |  |  |
| St. Louis River ^a^ | 6/5/2018 | 718 | 861 | 83.4 |
| Thomson Reservoir | 6/5/2018 | 671 | 833 | 80.6 |
| Embarrass River ^b^ | 6/6/2018 | 805 | 2906 | 27.7 |
| Embarrass Lake | 6/6/2018 | 642 | 984 | 65.3 |
| Pike River | 6/6/2018 | 929 | 1362 | 68.2 |
| Pike Bay, Lake Vermilion | 6/6/2018 | -- | 877 |  |
| Birch Lake inlet | 6/6/2018 | 207 | 385 | 53.7 |
| Birch Lake SW | 6/6/2018 | -- | 602 |  |
| Big Sandy River | 7/10/2018 | 2929 | 3282 | 92.6 |
| Big Sandy Lake | 7/10/2018 | 2159 | 2806 | 79.5 |
| Birch Lake inlet | 8/6/2018 | 370 | 466 | 79.2 |
| Birch outlet | 8/6/2018 | 531 | 682 | 77.9 |
| Embarrass Lake | 8/6/2018 | 2564 | 2791 | 91.9 |
| Embarrass River | 8/6/2018 | 3033 | 3367 | 90.1 |
| Johnson Lake | 8/5/2018 | 1788 | 1753 | 102.0 |
| Pike Bay, Lake Vermilion | 8/6/2018 | 1595 | 1851 | 86.2 |
| Pike River | 8/6/2018 | 2324 | 2584 | 89.9 |
| South Sturgeon | 8/5/2018 | 2356 | 2297 | 102.6 |
| Wolf Lake | 8/5/2018 | 3654 | 3793 | 96.3 |
|  |  |  |  |  |
| **(B) Averages** ^c^ | **N** |  |  |  |
| All data | 17-19 | 1604 | 1815 | 80.4 |
| Without Embar. Riv., 6/6 | 16-18 | 1654 | 1754 | 83.7 |
| Lakes only | 9 | 1773 | 1977 | 89.7 |
| Rivers only | 8 | 1414 | 1902 | 73.1 |
| Rivers w/o Embar. R., 6/6 | 7 | 1501 | 1758 | 79.6 |

^a^ Site upstream of Thomson Reservoir.

^b^ A large rain event the previous day resulted in very high flow and high turbidity in this sample, such that the inflow plume was visible hundreds of meters into the lake. Although Fe_T_ was even higher in this river in the Aug. 6 sample, it was mostly in dissolved form, similar to that for the lake into which the river drains. The low % Fe_diss_ for this sample thus likely represents an atypical situation.

^c^ Fe_T_ averages include the two lakes with no measured Fe_diss_; % Fe_diss_ averages are based on individual % Fe_diss_ values in part (A) and not on the ratio of the average Fe_diss_ and Fe_T_ values.
