# Supplementary material for: Iron influence on dissolved color in lakes of the Upper Great Lakes States
Source: PLoS One. 2019 Feb 13;14(2):e0211979. doi: 10.1371/journal.pone.0211979 (PMC6373958; doi:10.1371/journal.pone.0211979)
Supplement: S3 Table — (DOCX) [file pone.0211979.s006.docx]

**S3 Table**. Fe_diss_-*a*_440_ and Fe_diss_-DOC relationships for log-transformed data.

| **Year** | **N** | **Regression equation ^a^** | **R^2^** | **RMSE ^b^** | **Slope SE ^c^** |
| --- | --- | --- | --- | --- | --- |
| **Ln *a*_440_** | | |  |  |  |
| 2014 | 46 | ln(Fe_diss_) = 1.17×ln(*a*_440_) − 2.14 | 0.72 | 0.841 | 0.111 |
| 2015 | 61 | ln(Fe_diss_) = 1.284×ln(*a*_440_) − 2.69 | 0.81 | 0.681 | 0.081 |
| 2016 | 174 | ln(Fe_diss_) = 1.355×ln(*a*_440_) − 2.48 | 0.71 | 1.102 | 0.066 |
| All | 282 | ln(Fe_diss_) = 1.302×ln(*a*_440_) + 2.46 | 0.72 | 1.009 | 0.048 |
| **Ln DOC** | | |  |  |  |
| 2014 | 42 | ln(Fe_diss_) = 1.749×ln(DOC) − 0.248 | 0.59 | 0.981 | 0.225 |
| 2015 | 61 | ln(Fe_diss_) = 2.773×ln(DOC) − 2.485 | 0.74 | 0.794 | 0.214 |
| 2016 | 176 | ln(Fe_diss_) = 2.636×ln(DOC) − 2.34 | 0.51 | 1.415 | 0.195 |
| All | 280 | ln(Fe_diss_) = 2.515×ln(DOC) − 2.01 | 0.57 | 1.259 | 0.131 |

^a^ All regressions and coefficients significant at *p* < 0.0001.

^b^ Root mean square error.

^c^ Standard error of slope.
