# Supplementary material for: Iron influence on dissolved color in lakes of the Upper Great Lakes States
Source: PLoS One. 2019 Feb 13;14(2):e0211979. doi: 10.1371/journal.pone.0211979 (PMC6373958; doi:10.1371/journal.pone.0211979)
Supplement: S4 Table — (DOCX) [file pone.0211979.s007.docx]

**S4 Table.** **Log-transformed regression relationships for *a*_440_ vs. DOC and Fe_diss_.**

| **Data range** | **Best fit equation ^a^** | **N** | **R^2^** | **RMSE ^b^** | **Slope SE ^c^** |
| --- | --- | --- | --- | --- | --- |
| **All data** |  |  |  |  |  |
|  | ln(*a*_440_) = 2.043×ln(DOC) − 3.838 | 433 | 0.80 | 0.612 | 0.050 |
|  | ln(*a*_440_) = 0.558×ln(Fe_diss_) − 1.070 | 281 | 0.72 | 0.662 | 0.021 |
|  | ln(*a*_440_) = 1.351×ln(DOC) + 0.252×ln(Fe_diss_) − 3.057 | 276 | 0.89 | 0.422 | 0.068, 0.020 |
| ***a*_440_ > 3.0 m^-1^** | |  |  |  |  |
|  | ln(*a*_440_) = 1.528×ln(DOC) − 2.096 | 159 | 0.87 | 0.230 | 0.047 |
|  | ln *a*_440_ = 0.465×ln(Fe_diss_) − 0.328 | 136 | 0.65 | 0.381 | 0.030 |
|  | ln(*a*_440_) = 1.156×ln(DOC) + 0.168×ln(Fe_diss_) − 1.961 | 134 | 0.90 | 0.206 | 0.065, 0.023 |

^a^ All regressions and coefficients significant at *p* < 0.0001.

^b^ Root mean square error.

^c^ Standard error of slopes.
