# Supplementary material for: Iron influence on dissolved color in lakes of the Upper Great Lakes States
Source: PLoS One. 2019 Feb 13;14(2):e0211979. doi: 10.1371/journal.pone.0211979 (PMC6373958; doi:10.1371/journal.pone.0211979)
Supplement: S5 Table — (DOCX) [file pone.0211979.s008.docx]

**S5 Table. SUVA_254_ values for samples with measured SUVA_254_ > 5.0 before and after Fe_diss_ correction.**

| **Site** | **Sampling Date** | **Measured SUVA_254_ L mg^-1^ m^-1^** | **SUVA_254,DOM_ L mg^-1^ m^-1^** | **DOC mg/L** | **Fe_diss_ μg/L** |
| --- | --- | --- | --- | --- | --- |
| Blueberry Lake | 7/20/2016 | 5.20 | 4.97 | 26.9 | 978 |
| Johnson Lake | 9/23/2014 | 5.32 | 5.22 | 30.6 | 462 |
| Johnson Lake | 6/9/2015 | 5.84 | 5.69 | 25.1 | 589 |
| Johnson Lake | 7/13/2016 | 5.28 | 5.05 | 35.5 | 1279 |
| Johnson Lake | 8/3/2016 | 5.38 | 5.21 | 35.8 | 928 |
| Johnson Lake | 9/21/2016 | 5.24 | 5.00 | 33.0 | 1229 |
| Mineral Lake | 8/10/2016 | 5.80 | 5.52 | 25.1 | 1116 |
| Sabin Lake | 9/22/2014 | 5.04 | 4.91 | 22.4 | 433 |
| Big Sandy River L. | 9/15/2015 | 5.50 | 5.23 | 29.0 | 1217 |
| Section 11 Lake | 8/3/2016 | 5.29 | 5.14 | 32.4 | 783 |
| South Sturgeon Lake | 9/23/2014 | 5.25 | 5.14 | 30.7 | 502 |
| South Sturgeon Lake | 9/21/2016 | 5.07 | 4.84 | 30.9 | 1080 |
| Squaw Lake | 8/15/2016 | 5.47 | 5.44 | 15.5 | 72 |
| St. Louis River | 6/24/2014 | 5.31 | 5.26 | 26.3 | 214 |
| Vermilion Pike Bay | 8/3/2016 | 5.05 | 4.64 | 28.9 | 1858 |
