# Supplementary material for: Iron influence on dissolved color in lakes of the Upper Great Lakes States
Source: PLoS One. 2019 Feb 13;14(2):e0211979. doi: 10.1371/journal.pone.0211979 (PMC6373958; doi:10.1371/journal.pone.0211979)
Supplement: S6 Table — (DOCX) [file pone.0211979.s009.docx]

**S6 Table. Changes in *a*_440_ for 2015 waters that had large differences between measured and best-fit Fe_diss_ after Fe_diss_ was changed to the best-fit value.**

| **Lake ^a^** | **Measured *a*_440_ m^-1^** | **Measured Fe_diss_ μg/L** | **Best-fit Fe_diss_ μg/L** | **Change in *a*_440_ m^-1^** | **Fe-corrected *a*_440_, m^-1^** | **% change in *a*_440_** |
| --- | --- | --- | --- | --- | --- | --- |
| Blueberry L. (8/17) | \| 19.6 \| \| --- \| | 1224 | 690 | −1.3 | 18.3 | −6.6 |
| Big Sandy River L. | 23.3 | 1217 | 825 | −1.0 | 22.3 | −4.3 |
| South Sturgeon (6/10) | 17.0 | 731 | 597 | −0.3 | 16.7 | −1.8 |
| Johnson StL. | 13.4 | 737 | 461 | −0.7 | 12.7 | −5.2 |
| Big Sandy L. | 11.5 | 658 | 393 | −0.6 | 10.9 | −5.2 |
| Shoepack L. | 9.4 | 491 | 315 | −0.4 | 9.0 | −4.2 |
| Thomson Res. (9/15) | 24.2 | 577 | 860 | +0.7 | 24.9 | +2.9 |
| Johnson L. It. (6/9) | 23.5 | 589 | 834 | +0.6 | 24.1 | +2.5 |
| Johnson L. It. (9/15) | 20.0 | 492.5 | 707.1 | +0.5 | 20.5 | +2.5 |
| Section 11 L. (6/9) | 18.6 | 446.7 | 656.0 | +0.5 | 19.1 | +2.7 |
| Thomson Res. (6/10) | 16.3 | 423.8 | 571.3 | +0.4 | 16.7 | +2.4 |
| Blueberry L. (6/24) | 16.1 | 466.6 | 562.8 | +0.2 | 16.3 | +1.2 |
| So. Sturgeon L. (9/15) | 14.7 | 395.8 | 512.0 | +0.3 | 15.0 | +2.0 |
| Section 11 L. (9/15) | 14.0 | 387.2 | 486.6 | +0.2 | 14.2 | +1.4 |

^a^ Lakes with dates in parentheses were sampled multiple times. Abbreviations indicate county location for two lakes named Johnson: It. = Itasca; StL. = St. Louis.
