# Supplementary material for: Iron influence on dissolved color in lakes of the Upper Great Lakes States
Source: PLoS One. 2019 Feb 13;14(2):e0211979. doi: 10.1371/journal.pone.0211979 (PMC6373958; doi:10.1371/journal.pone.0211979)
Supplement: S7 Table — (DOCX) [file pone.0211979.s010.docx]

**S7 Table. Samples with measured *a*_440_ > 3.0 m^-1^ having 15-30% of *a*_440_ caused by Fe_diss_ and 70-85% caused by colored DOM.**

| **Lake** | **State** | **Date** | **DOC mg/L** | **Fe_diss_ μg/L** | **Meas. *a*_440_ m^-1^** | **Fraction of *a*_440_ due to DOM** |
| --- | --- | --- | --- | --- | --- | --- |
| Big Sandy Lake | MN | 6/8/16 | 15.4 | 840 | 9.9 | 0.795 |
| Big Sandy Lake | MN | 8/4/16 | 20.3 | 897 | 12.7 | 0.829 |
| Big Sandy Lake | MN | 9/26/16 | 20.1 | 877 | 10.8 | 0.804 |
| Big Sandy River Lake | MN | 7/12/16 | 20.7 | 1584 | 13.4 | 0.713 |
| Clark Lake | MI | 8/23/16 | 8.3 | 1404 | 11.4 | 0.713 |
| Rainbow Flowage | WI | 9/24/16 | 10.1 | 584 | 5.8 | 0.755 |
| Lake. St. Croix | MN | 10/7/16 | 10.0 | 391 | 5.1 | 0.813 |
| Lake Vermilion,  Pike Bay | MN | 8/3/16 | 28.9 | 1858 | 24.2 | 0.814 |
| Wolf Lake | MN | 9/13/16 | 31.4 | 1646 | 22.1 | 0.820 |
